# Outcomes of Patients Requiring Blood Pressure Control Before Thrombolysis with tPA for Acute Ischemic Stroke

**DOI:** 10.5811/westjem.2015.8.27859

**Published:** 2015-12-16

**Authors:** Bryan Darger, Nicole Gonzales, Rosa C. Banuelos, Hui Peng, Ryan P. Radecki, Pratik B. Doshi

**Affiliations:** * University of Texas Medical School at Houston, Houston, Texas; † University of Texas Health Science Center at Houston, Department of Emergency Medicine, Houston, Texas; ‡ University of Texas Health Science Center at Houston, Department of Neurology, Houston, Texas

## Abstract

**Introduction:**

The purpose of this study was to assess safety and efficacy of thrombolysis in the setting of aggressive blood pressure (BP) control as it compares to standard BP control or no BP control prior to thrombolysis.

**Methods:**

We performed a retrospective review of patients treated with tissue plasminogen activator (tPA) for acute ischemic stroke (AIS) between 2004–2011. We compared the outcomes of patients treated with tPA for AIS who required aggressive BP control prior to thrombolysis to those requiring standard or no BP control prior to thrombolysis. The primary outcome of interest was safety, defined by all grades of hemorrhagic transformation and neurologic deterioration. The secondary outcome was efficacy, determined by functional status at discharge, and in-hospital deaths.

**Results:**

Of 427 patients included in the analysis, 89 received aggressive BP control prior to thrombolysis, 65 received standard BP control, and 273 required no BP control prior to thrombolysis. Patients requiring BP control had more severe strokes, with median arrival National Institutes of Health Stroke Scale of 10 (IQR [6–17]) in patients not requiring BP control versus 11 (IQR [5–16]) and 13 (IQR [7–20]) in patients requiring standard and aggressive BP lowering therapies, respectively (p=0.048). In a multiple logistic regression model adjusting for baseline differences, there were no statistically significant differences in adverse events between the three groups (P>0.10).

**Conclusion:**

We observed no association between BP control and adverse outcomes in ischemic stroke patients undergoing thrombolysis. However, additional study is necessary to confirm or refute the safety of aggressive BP control prior to thrombolysis.

## INTRODUCTION

Acute ischemic stroke (AIS) is a major cause of morbidity and mortality. AIS affects over 15 million patients yearly worldwide, and represents the fifth-leading cause of death and leading cause of disability in the United States.^1^ Currently, the only FDA-approved medical therapy for treatment of AIS is thrombolysis with recombinant tissue plasminogen activator (tPA) within three hours of symptom onset. However, despite this being the only approved medical therapy, the majority of eligible patients remain untreated. One of the reasons for this under-treatment stems from exclusion of patients who present with elevated blood pressure (BP).^2^–^4^ Prior literature has found that patient BPs in excess of the pre-thrombolytic goal of 185/110 is associated with delayed^5^ and non-treatment with thrombolytics.^6^ and that active management of BP in these patients is associated with an increased proportion receiving thrombolytic therapy.^7^

The original National Institute for Neurologic Disorders and Stroke rt-PA Stroke Study (NINDS) excluded patients with BP>185/110, as well as those requiring aggressive BP lowering prior to thrombolysis. The rationale was an observed association between this level of hypertension and increased risk of intracranial hemorrhage after thrombolysis, largely extrapolated from the cardiac literature and the NINDS tPA pilot study.^8^–^10^ What constituted aggressive treatment was not specifically defined in the NINDS protocol but has generally been considered to include continuous infusion of antihypertensive medication or repeated doses of antihypertensive medications, such as labetalol, enalapril, nicardipine, or nifedipine.^7^

Since the original NINDS trial, many versions of guidelines and protocols have excluded these patients from eligibility for treatment with tPA.^11^,^12^ However, current evidence does not consistently observe an association between elevated BP and adverse outcomes in patients treated with thrombolytics.^2^,^13^,^14^ While the latest guidelines from the American Heart Association regarding the treatment of stroke allow for use of intravenous (IV) anti-hypertensive therapy previously considered aggressive, this is largely based on expert opinion due to a paucity of evidence regarding the treatment of arterial hypertension in the setting of stroke.^15^ A recent post hoc analysis of two large randomized controlled trials found 21% of 1,657 AIS patients who were otherwise eligible for IV thrombolysis had elevated pretreatment BP above 185/110mmHg,^7^ a figure consistent with previous retrospective studies.^3^ It is clear that further evidence is needed to determine the optimal management of these patients.

In this study, we aim to further assess the safety and efficacy of thrombolysis in the setting of aggressive BP control as it compares to standard BP control or no BP control prior to thrombolysis.

## METHODS

This was a retrospective analysis of registry data from the University of Texas Health Science Center at Houston, collected between 2004 and 2011.^16^ We reviewed all patients who were treated with IV tPA for AIS within 4.5 hours of symptom onset. Patients treated with anti-hypertensive medications other than labetalol or nicardipine, treated beyond 4.5 hours, or who were enrolled in clinical trials were excluded.

Data collected included time of symptom onset and patient arrival, all recorded vital signs, clinical findings, baseline computed tomographic findings, comorbidities, medication history, dosage and time of administration of antihypertensive agents in the emergency department, and time of tPA administration. BP and the type and dosage of anti-hypertensive medications used were abstracted with an additional chart review by two independent abstractors. Long-term outcome data, such as pre-stroke functional status and 90-day clinical outcome measures, were available only for a limited number of patients and therefore were not included as part of the analysis.

Aggressive BP control was defined as continuous nicardipine infusion or greater than two doses of IV labetalol, whereas standard BP control was defined as requiring two or less doses of IV labetolol prior to thrombolysis. The primary outcome was safety as measured by all grades of hemorrhagic transformation, symptomatic intracranial hemorrhage, and neurologic deterioration. The secondary outcome was efficacy as measured by good functional status at hospital discharge, hospital inpatient length of stay, and in-hospital deaths. Symptomatic intracerebral hemorrhage was defined as parenchymal hematoma likely to be the cause of neurologic deterioration. Good functional status at hospital discharge was defined as a score of 0 to 2 on the modified Rankin Scale (mRS).

Demographic variables and baseline characteristics are described by the median and interquartile range (IQR) or count and percentage for continuous and categorical variables, respectively. We carried out comparisons between groups using the Kruskall-Wallis test for continuous variables and the chi-square test for categorical variables. To compare outcomes between groups, multiple logistic regression models were fit adjusting for certain demographic and/or baseline characteristic variables (see Table 2 for details). Expressing hospital length of stay (HLOS) as time to discharge, we applied a Cox proportional hazards (PH) model with right censoring to account for death during hospital stay. The number of variables included in the models was restricted by to the limited sample size and thus variables deemed clinically meaningful were included. We reported adjusted odds ratios (OR) and the adjusted hazard ratio (HR) with corresponding 95% confidence interval (CI) for the multiple logistic regression models and the Cox PH model, respectively. Statistical significance was assessed at the 5% significance level. We analyzed the data using the SAS 9.4 statistical software (SAS Institute, Inc., Cary, NC).

This study was approved by the institutional review board of the University of Texas, which granted a waiver of informed consent for this retrospective review.

## RESULTS

Of the 427 patients included in the analysis, 273 patients did not require BP control prior to thrombolysis with tPA, while 65 required standard BP treatment and the remaining 89 required aggressive BP control prior to thrombolysis. The median age in the no BP and standard BP treatment groups were 67 and 68 years, respectively while those in the aggressive BP treatment group had a median age of 72. There were more males in the no BP (57%) and standard BP (54%) treatment groups in comparison to 43% males in the aggressive BP treatment group. Overall, the patients were similar in demographic and baseline characteristics, with the exception of National Institutes of Health Stroke Scale (NIHSS) where the group requiring aggressive BP control had a greater median arrival NIHSS of 13 (IQR [7–20]) as compared to the no BP and standard BP treatment groups (see details in Table 1). Also noteworthy was that patients not requiring BP control received thrombolytic therapy in similar timeframes to patients requiring BP control.

In patients receiving aggressive BP control 8% had hemorrhagic complications and 23% had neurologic complications during their hospitalization. However, when adjusting for other covariates, these differences were not statistically significant for both neurologic deterioration (p=0.554) and hemorrhagic transformation (p=0.156).

After adjusting for arrival NIHSS, age, and gender, the odds of in-hospital mortality were not different in patients requiring either type of BP control prior to thrombolysis when compared to patients not requiring BP control (OR 0.70, 95% CI [0.22–2.29] for standard therapy, OR 1.27, 95% CI [0.56–2.89] for aggressive therapy, p=0.649).

Regarding discharge functional status for survivors as measured by a mRS of 0–2, after adjustment for differences in baseline characteristics, no significant differences were observed between patients requiring either BP-lowering strategies as compared to patients not requiring BP control prior to thrombolysis (OR 1.14, 95% CI [0.60–2.15] for standard therapy, OR 0.56, 95% CI [0.29–1.07] for aggressive therapy, p=0.157). Detailed outcomes are reported in Table 2.

In comparing HLOS between the three groups, after adjusting for age, gender, glucose, and arrival NIHSS, there were no significant differences in HLOS between these groups (HR 0.67, 95% CI [0.22.-2.02], for standard therapy, HR 0.82, 95% CI [0.37–1.84], for aggressive therapy, p=0.732; see Table 3)

## DISCUSSION

Our study represents a large data set comparing patients requiring BP control prior to thrombolysis to those who did not. There were a few important findings of note. The primary finding of our study is that patients presenting with very elevated BPs in the setting of AIS generally have more severe strokes and are older, and after correcting for these baseline differences, treatment with thrombolysis does not seem to add additional risk of hemorrhage in patients requiring BP control as compared to patients not requiring BP control prior to thrombolysis.

Previous literature supports our finding that patients presenting with AIS who have an extremely elevated BP have worse outcomes, which is likely due to the baseline increased severity in stroke symptoms.^17^,^18^ Because there is a very limited amount of literature addressing this question, our study represents the largest experience with pre-treatment BP control in the setting of thrombolysis in AIS. Since in our study, we could not detect a statistically significant difference in adverse events between patients requiring any method of BP control prior to thrombolysis versus those who did not, when adjusting for the baseline severity of the stroke, it is possible that patients presenting with elevated BP in the setting of AIS who may otherwise be eligible for thrombolysis could be considered for thrombolysis after optimization of BP.

The second important finding of our study is that the management of elevated BP prior to thrombolysis did not significantly increase door-to-tPA time. This is an improvement over the temporal impact of antihypertensive treatment described in previous literature.^5^ This underlines how, with an appropriate process in place, door-to-tPA time may yet be optimized even in those patients requiring further interventions prior to initiation of therapy. As has been the case with other performance improvement strategies used for decreasing the door-to-tPA time in AIS, such as pre-hospital notification^19^, direct transport to computed tomography,^20^ tPA availability in the emergency department and systemized activation of treatment teams,^21^ the utility of antihypertensive agents to achieve desired BP control prior to thrombolysis can be incorporated in routine stroke care. An example of such a process using rapidly acting agents to achieve pre-treatment goals in patients with AIS was recently described in the literature by Bowry et al.^22^

## LIMITATIONS

There are several limitations to this study. This is a retrospective review of prospectively collected data, which may be useful for hypothesis generation but is not well-suited for evaluating the safety or efficacy of a clinical intervention. Additional, unmeasured, prognostic variables may confound our adjusted statistics. Our registry does not provide details regarding the selection criteria by which patients were chosen for treatment with thrombolysis after BP control, nor do we report data regarding patients presenting with hypertension in the setting of AIS who did not receive treatment with tPA. In addition, it does not provide details of the amounts and types of medications used for BP optimization. Functional outcomes are also available only at hospital discharge, rather than the three- to six-month follow up typical to stroke research. Lastly, the results of this study represent the experience of a single, high-volume academic stroke center over a seven-year time period, and may not be generalizable to other institutions.

Given the totality of evidence, the limitations of our study preclude a declaration of safety associated with BP control prior to thrombolysis; however, it does underline the need for further study to allow for up to 25% of otherwise-eligible patients to be considered for the only approved therapy for AIS.

## CONCLUSION

This study represents the largest data set to date evaluating the outcomes experienced by patients requiring BP control prior to receiving treatment for ischemic stroke with tPA in the emergency department. In the context of prior research suggesting harms associated with BP treatment prior to thrombolysis, these data suggest it may be reasonable to further investigate subgroups of patients for whom BP pretreatment does not confer additional risk. In addition, these data also show, with integrated efforts, the treatment of elevated arterial BP need not be associated with significantly longer door-to-tPA time, as has been previously reported.^5^,^15^

## Figures and Tables

**Table 1:** Summary of demographic variables and baseline characteristics between groups.

Variable	No BP treatment before tPA (n=273)	Standard BP treatment before tPA (n=65)	Aggressive treatment before tPA (n=89)	P-value
Age, median (IQR)	67 (55–80)	68 (57–80)	72 (59–82)	0.227
Male gender, n (%)	156 (57.1)	35 (53.9)	38 (42.7)	0.060
White race, n (%)	175 (64.1)	41 (63.1)	52 (58.4)	0.629
Latino ethnicity, n (%)	44 (16.2)	8 (12.5)	8 (9.0)	0.218
Arrival NIHSS score, median (IQR)	10 (6–17)	11 (5–16)	13 (7–20)	0.048
Glucose, median (IQR)	122 (106–154)	118 (99–146)	126 (108–155)	0.234
Initial systolic BP, median (IQR)	152 (137–168)	175 (155–185)	180 (165–194)	-
Initial diastolic BP, median (IQR)	80 (71–89)	89 (79–98)	89 (81–99)	-
Time from door to tPA (min), median (IQR)	69 (51–88)	68 (53–82)	76 (58–95)	0.146
Time from onset to tPA (min), median (IQR)	149 (121–175)	150 (122–175)	157 (130–182)	0.407
History of hypertension, n (%)	192 (70.3)	50 (76.9)	73 (82.0)	0.077
Patients receiving long-term BP medication, n (%)	155 (65.1)	35 (71.4)	53 (67.1)	0.689

*BP,* blood pressure; *tPA,* tissue plasminogen activator; *IQR,* interquartile range; *NIHSS,* National Institutes of Health Stroke Scale

**Table 2:** Analysis of outcome variables and group via multiple logistic regression.

Outcome variable	Comparison to baseline*	AOR (95% CI)	P-value
Neurologic deterioration†	Standard BP treatment before tPA	0.71 (0.33, 1.54)	0.554
	Aggressive treatment before tPA	1.15 (0.63, 2.11)	
Hemorrhagic transformation	Standard BP treatment before tPA	1.04 (0.22, 5.04)	0.156
	Aggressive treatment before tPA	2.71 (0.95, 7.76)	
Symptomatic hemorrhage‡	Standard BP treatment before tPA	0.69 (0.15, 3.15)	0.889
	Aggressive treatment before tPA	0.97 (0.30, 3.10)	
Good outcome§	Standard BP treatment before tPA	1.14 (0.60, 2.15)	0.157
	Aggressive treatment before tPA	0.56 (0.29, 1.07)	
Death||	Standard BP treatment before tPA	0.70 (0.22, 2.29)	0.649
	Aggressive treatment before tPA	1.27 (0.56, 2.89)	

*AOR*, adjusted odds ratio

* Baseline set as no blood pressure (BP) treatment before tissue plasminogen activator (tPA).

† Adjusted for age, gender, glucose, arrival National Institutes of Health Stroke Scale (NIHSS) score, time from onset to tPA (min).

‡ Adjusted for arrival NIHSS score.

§ Adjusted for age, gender, glucose, arrival NIHSS score, time from onset to tPA (min).

|| Adjusted for age, gender, arrival NIHSS score.

**Table 3:** Hazard ratios (HR) for length of hospital stay (time to discharge) via Cox PH model.

Outcome variable	Comparison to baseline	HR* (95% CI)	P-value
Length of hospital stay	Standard BP treatment before tPA	0.67 (0.22, 2.02)	0.732
	Aggressive treatment before tPA	0.82 (0.37, 1.84)	

*BP,* blood pressure; *tPA,* tissue plasminogen activator; *PH,* proportional hazards

* Adjusted for age, gender, glucose, and arrival National Institutes of Health Stroke Scale score.
